# Supplementary material for: 5′RNA Trans-Splicing Repair of COL7A1 Mutant Transcripts in Epidermolysis Bullosa
Source: Int J Mol Sci. 2022 Feb 2;23(3):1732. doi: 10.3390/ijms23031732 (PMC8835740; doi:10.3390/ijms23031732)
Supplement: Supplementary file 1 [file ijms-23-01732-s001.zip › ijms-1487539-supplementary.pptx]

## Slide 1
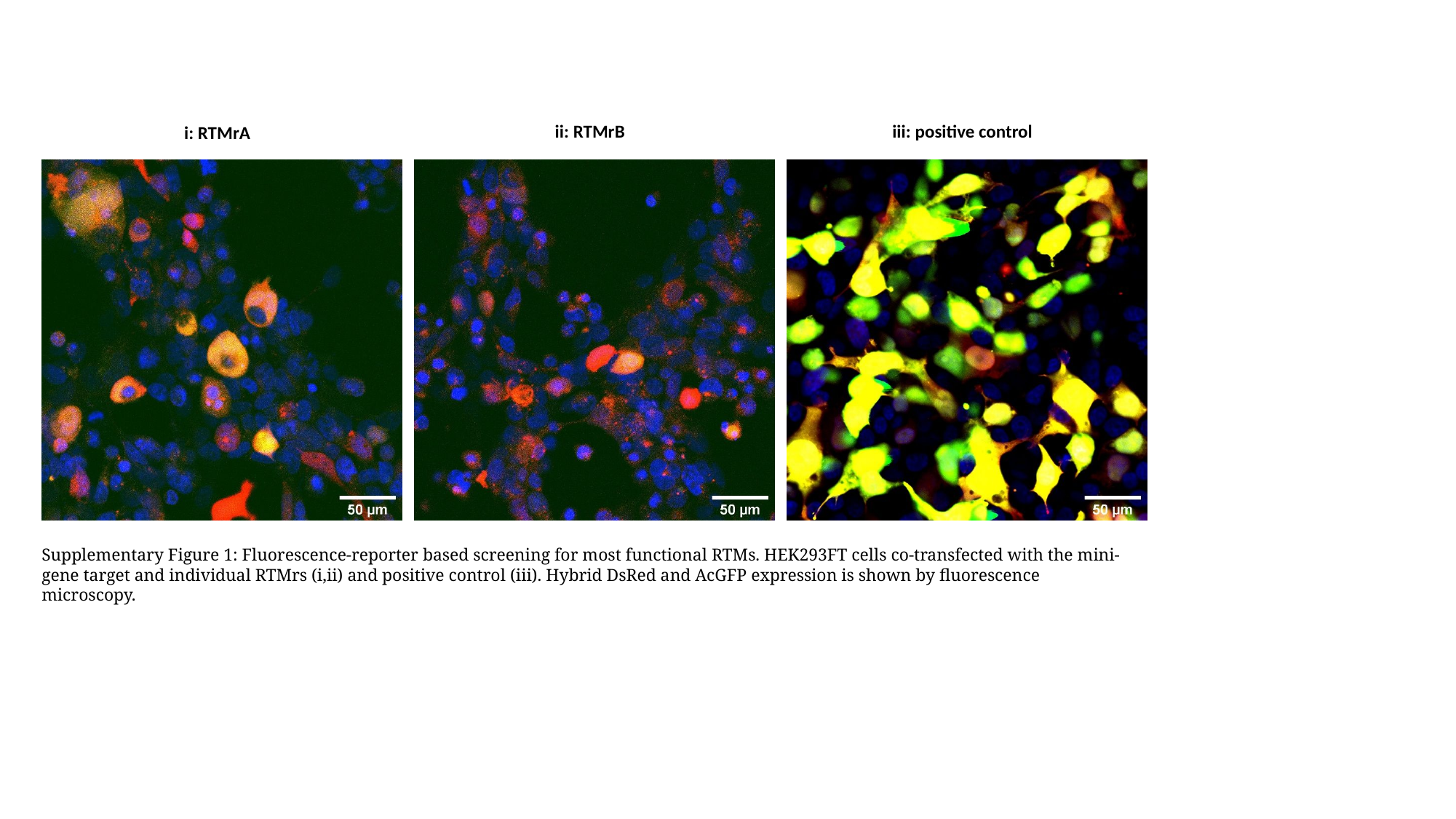

iii: positive control
ii: RTMrB
i: RTMrA
Supplementary Figure 1: Fluorescence-reporter based screening for most functional RTMs. HEK293FT cells co-transfected with the mini-gene target and individual RTMrs (i,ii) and positive control (iii). Hybrid DsRed and AcGFP expression is shown by fluorescence microscopy.

## Slide 2
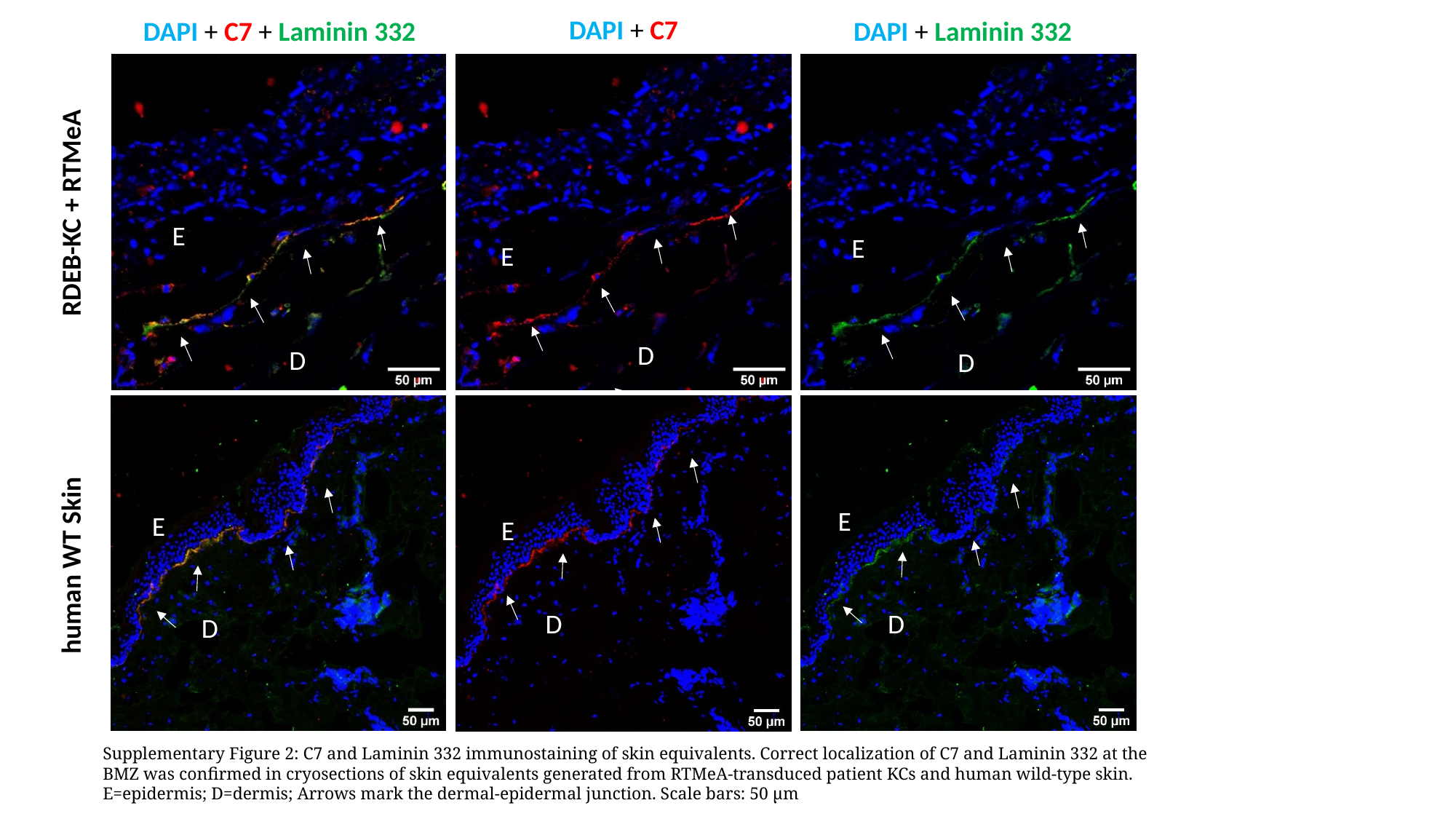

DAPI + C7
DAPI + C7 + Laminin 332
DAPI + Laminin 332
RDEB-KC + RTMeA
E
E
E
D
D
D
E
E
E
human WT Skin
D
D
D
Supplementary Figure 2: C7 and Laminin 332 immunostaining of skin equivalents. Correct localization of C7 and Laminin 332 at the BMZ was confirmed in cryosections of skin equivalents generated from RTMeA-transduced patient KCs and human wild-type skin. E=epidermis; D=dermis; Arrows mark the dermal-epidermal junction. Scale bars: 50 µm
